# Who is watching? Refugee protection during a pandemic - responses from Uganda and South Africa

**DOI:** 10.1186/s40878-021-00243-3

**Published:** 2021-08-10

**Authors:** Khangelani Moyo, Kalyango Ronald Sebba, Franzisca Zanker

**Affiliations:** 1 Global Change Institute, University of Witwatersrand, 1 Jan Smuts Avenue, Johannesburg, 2001 South Africa; 2 School of Women and Gender Studies, Pool Road Makerere University, Kampala, Uganda; 3 Arnold Bergstraesser Institute at the University of Freiburg, Windausstr. 16, 79110 Freiburg, Germany

**Keywords:** Refugees, Borders, COVID-19, UNHCR, Xenophobia, Securitisation, Host communities, Social protection

## Abstract

Both Uganda and South Africa were quick to respond to the global pandemic – Uganda for example imposing quarantine on foreign travellers after only a handful of cases before shutting off all international flights, and South Africa imposing one of the first lockdowns on the continent. Reflecting on the first 6 months of the pandemic responses in terms of refugee protection, the two countries have taken diverging pathways. South Africa used the pandemic to start building a border fence on the border with Zimbabwe, initially curtailed all foreign shop owners from opening under lockdown and excluded asylum seekers from emergency relief grants. In contrast, Uganda opened its borders to refugees from the DRC in June, when border closures were still the global norm. Whilst both responses are not unusual in light of their standard governance approaches, they highlight the own self-image the countries espouse – with Uganda positioning itself as the world’s premier refugee protector at a time when it is desperately in need of more funds and South Africa looking to politically capitalize internally from reiterating a division between migrant communities as a threat to poor and disenfranchised South Africans. Even during a pandemic, the practice of refugee protection does not happen in a political vacuum. This paper is based on over 50 in-person and digital interviews conducted in Uganda and South Africa in 2020, as well as nine focus groups with refugee and host communities.

## Introduction

By the time the WHO officially declared the novel COVID-19 virus a pandemic – on the 11th March – both Uganda and South Africa were extremely quick to respond. On the 18th March, before a single case of the virus was confirmed in Uganda, all Ugandans and foreign nationals arriving in the country were put into a mandatory two-week quarantine with the rest of the country placed under a strict lockdown shortly after. Travellers from countries at high risk – at the time all those over 100 cases - were banned completely, for 32 days initially. In a press statement at the time president Museveni said “Foreigners going to those countries are free to do so provided they do not intend to come back within the prohibited time. We extend our sympathies to those countries and commend them for fighting on behalf of the human race” (Ssebwami, 2020). The airport only re-opened 6 months later, in October, allowing all those with a negative COVID-19 test result from the last 72 h to enter the country.

In South Africa, President Ramaphosa announced a state of disaster on the 15th March, with travel restrictions and school closures to start 2 days later. Like in Uganda, there was a travel ban for all foreign nationals from high-risk countries, with visas cancelled. Out of the 72 ports of entry, 35 land ports and two sea ports were shut down (Republic of South Africa, 2020). From the 26th March, the government imposed one of the world’s strictest lockdowns by which time the country had 927 cases of coronavirus. International tourists were welcomed back by the 1st of October 2020, with the exception of specified countries with high rates of active infections, including the USA and the UK.

In both countries, the lockdown measures have had extreme repercussions. In South Africa, a decline of at least 7% in the GDP was expected for 2020, with unemployment levels already at 30% even prior to the lockdown measures (Jazeera, 2020). In Uganda, the GDP was still expected to grow in 2020, albeit at a much slower pace than what had been predicted prior to the pandemic outbreak (Focus Economics, 2020). Nonetheless, given that much of the economy is built on the informal working sector, the real impact will take a longer time to become clear (see also Hartwig & Lakemann, 2020). This economic shortfall also affects refugee protection, as will be discussed further below.

How does the pandemic affect arguably some of the most vulnerable populations, namely refugees and other migrants?^1^ And how does the pandemic response link back to pre-existing political interests when it comes to protecting refugees? In the following, we compare the two countries – that not only differ in their size, economic power and geographic positioning, but also how they govern refugees and migration more broadly.

Theoretically, the paper builds on the premise that refugee hosting can add to domestic and international legitimacy in different ways. Domestically, refugee hosting is often tied up in questions of citizenship and belonging, where governments may seek out political capital by excluding newcomers on the basis of belonging (e.g. Nyamnjoh, 2006), or as a security threat (e.g. Jaji, 2014; Okyerefo & Setrana, 2018). This is often done in the face of competition over access to public goods between refugees and host communities (Whitaker, 2019). Internationally, refugee hosting can be tied into seeking leverage with donors (e.g. Milner, 2009; Whitaker, 2017) or other forms of so-called “migration diplomacy” (e.g. Adamson & Tsourapas, 2019).

In order to discuss the internal and external leverage we will introduce some of the characteristics of refugee protection in South Africa and Uganda, before showing the respective pandemic responses. We discuss borders, social protection of refugees and the public discourse around refugee protection respectively. In a final section we compare the two countries’ responses, before concluding. We argue that their pandemic responses towards refugees align with their wider goals – gaining legitimacy from a frustrated public (South Africa) versus cementing a positive image for the international community, not least in the wake of the elections in January 2021 (Uganda).

In South Africa, (already pre-pandemic) high unemployment, inequality, poverty and corruption has put the ruling party on a stress test. Exacerbated by the fact that the parliamentary majority of the ruling Africa National Congress reduced in the 2019 elections, from 61.15% in 2014 to 57.50%, making finding a scapegoat for many of the problems in the country of interest. In Uganda, the elections held in January 2021 were hotly contested, with clampdowns on politicians and journalists receiving international condemnation (United Nations, 2020). The outcome of this election is beyond the timeframe of this paper, which focuses on the first 6 months of the pandemic, but already in 2020 the elections were building up to be the most contested in the 35-year rule of President Museveni (Kamoga, 2020).

The paper is based on research from an ongoing project which considers the political stakes of refugee and migration governance in the two countries.^2^ The research employed semi-structured interviews and focus group discussions with both refugee and host community groups. Fieldwork in South Africa took place in Johannesburg and Musina in February and March 2020, ending shortly before the lockdown was imposed. Fieldwork in Uganda has been delayed due to COVID-19, with digital interviews and some restricted fieldwork going on at the time of writing (September – October 2020) in Arua and Kampala. In total, this paper draws on 32 interviews in South Africa (both in person and online) and 24 interviews in in Uganda (mainly online). Interviewees include policy makers, politicians, civil society activists, diaspora leaders and academic experts. Whilst digital and telephone interviews do not allow for the same level of exchange in a trustful environment as face-to-face ones, since they were conducted at a time when the pandemic increasingly become the “new normal” (April–October 2020), participants were more open to them as they might have been otherwise. The research also considers nine focus groups with refugee and host communities conducted in Kampala, Johannesburg, Musina and Arua. The focus groups in Uganda were conducted with adherence to health measures in light of COVID-19, including temperature checks, mask provisions, hand washing facilities and social distancing.

The paper will first discuss the characteristics of refugee protection in South Africa and Uganda respectively, before the pandemic response in the two countries further below.

### Characteristics of refugee protection

In South Africa it is notoriously difficult to get figures on migrants and refugees, having long been overblown for political gains. By 2019, there were 188,296 pending asylum seeker applications in the country (UNHCR, 2020a). According to an audit of the Department of Home Affairs (DHA; in charge of all migrant and refugee affairs), at the current speed of asylum appeals it would take 68 years to work through the current backlog (Auditor General of South Africa, 2019).

More generally, South African refugee protection follows a logic of local integration. Asylum-seekers – upon receiving the right papers (Section 22 permit, see below) – are free to move around the country, work and study as they please. Whilst this makes it one of the most generous refugee systems in the world, it is marred in practice by a lack of implementation of these regulations and an increasingly restrictive regime curtailing all these rights (Amnesty International, 2019; Zanker & Moyo, 2020). Two major characteristics underline the refugee protection system in South Africa both tied to the domestic legitimacy gains through exclusion as introduced above: a state-led bureaucratized merging of refugees and migrants, in the shadow of institutionalized xenophobia leading to increasing securitisation.

On the first point, due to a number of changes in refugee laws with recent amendments, asylum seekers are faced with increasing and excessive bureaucratic processes, that effectively curtail their rights. Refugees need to formally enter the country in order to get a transit visa. They then have 5 days to report to a Refugee Reception Office (RRO), under the authorities of the DHA. RROs have nationality days, which means only certain nationalities can report on certain days, already highlighting one of the unfeasibility of the current system. Pending their hearings, asylum seekers are given a temporary – so-called section 22 permit - which they have to renew every few months, but sometimes after only a few weeks. Under the new regulations, asylum claims are considered to be ‘abandoned’ if the section 22 permits are not renewed for more than 30 days, leaving asylum seekers subject to deportation potentially at the cost of violating *non-refoulement* standards of refugee protection. Further complicating matters, between 2011 and 2020 only half of the six RROs were fully functioning, with others fully or partially closed despite repeated court orders (see also Estifanos et al., 2019). Continuously having to renew permits is burdensome, and carries many economic and psycho-social costs for asylum seekers. The “paper wall” adds to the burdens many asylum seekers carry, many living in dire conditions. In a focus group with local residents in the border town of Musina, one participant noted “the government is not protecting refugees because if we look at places where refugees stay, you can tell it’s not safe, not protected” (Musina, 7th March 2020).

Since there are limited opportunities for low-skilled migrant workers to live in South Africa legally, many look for other ways to regulate their stay, including through a section 22 asylum permit. This misuse of the asylum system by economic migrants is often highlighted by the government as the cause of inefficiencies and the insurmountable backlog in processing (Amnesty International, 2019). How frequently this actually takes place however or how to provide long-term alternatives for legalisation is not addressed in a transparent manner, and the situation remains open to xenophobic co-optation.

On this second point, xenophobic violence against migrant and refugee communities continues routinely, with a lack of political will to address xenophobia at best, and complicity amongst a spectrum of politicians and civil servants including the police at worst (see also Misago, 2019; Neocosmos, 2008). Beyond actual physical violence, there is also institutional xenophobia which continue to pervade in South Africa. From hospitals to schools to the institutions of the DHA there is systemic and institutionalised xenophobia, which makes the lives of many refugees and other migrants particularly difficult. Lastly, tied to the xenophobic violence, South Africa follows a very securitized trend. Though this has antecedents in the previous apartheid regime (e.g. Musoni, 2020), it is also a recent development that builds on what is otherwise a very progressive framework for protecting refugees. A White Paper on International Migration from 2017 (which includes refugees) notes that the government wants “South Africa to adequately embrace global opportunities while safeguarding our sovereignty and ensuring *public safety* and *national security*”, highlighting they want to pursue a ‘risk-based approach’ in migration governance (Department of Home Affairs, 2017, pp. 2–3). One example of this securitized response around this time is the heavy-handed, militaristic Operation Fiela (or “sweep clean”), which was launched in response to xenophobic violence in 2015 (with a second round in 2018). Instead of arresting perpetrators of xenophobic violence it rounded up undocumented migrants, violating a number of legal and human rights provisions (Dodson & Crush, 2015). In July 2020, a new Border Management Authority Act was signed into law by President Ramaphosa. The Act has long been in the making, but faces criticism as undermining free movement, for potentially abusing basic principles of refugee protection and painting a militarized picture of ‘migrant invasion’ (Bornman, 2020a). According to one academic: “… managing immigration … can be seen increasingly as security issues and more specifically national security issues rather than just social problems” (interview, Johannesburg, February 2020).

The picture in Uganda offers a contrast. As of 31 August 2020, there were 1,429,268 refugees and asylum seekers in Uganda (UNHCR, 2020d). These are spread in eleven refugee settlements in 12 districts (refugees in the capital Kampala are not in a settlement). All refugees – like Ugandans – have access to healthcare and subsidised primary education (e.g. Ruzibiza et al., 2021), guaranteed along the other rights in Refugee Act from 2006 and the accompanying Regulations of 2010. This Act has been named the “most progressive refugee law in Africa” (ODI, 2019).

Most refugees are from South Sudan or DRC (61.7% and 29.3%, respectively), and they get prima facie refugee status under the Organisation of African Union Refugee Convention of 1969. Other refugees – like from Burundi, Rwanda or Eritrea – have to go claim for asylum and thus go through the formal status determination procedures. Uganda has long adopted a development approach towards refugees, whereby they aim to make the refugee population less dependent on humanitarian aid in the long run, as well as incorporate the host population in a more integrative development approach. This has been pursued by a variety of approaches, including the Self-Reliance Strategy (from 1999); the Development Assistance to Refuge-Hosting Areas Programme (2004) and the Settlement Transformative Agenda launched in 2015. The Transformative Agenda underpinned that Uganda was pursuing a non-encampment approach and that refugees are part of the broader development agenda of the country. In the same year, refugees were included in the National Development Plan II (NDP) and the ReHoPE Strategic Framework was introduced. This brought in a “30–70” principle, whereby at least 30% of all interventions for refugees should target host-community needs.

Finally, as a follow-up to the New York Declaration of Refugees and Migrants, the Comprehensive Refugee Response Framework (CRRF), incorporated into the Global Compact on Refugees, was launched in 2017. A multi-stakeholder coordination model on refugee matters focusing on humanitarian and development needs of both refugees and host communities, the CRRF was introduced in Uganda, Ethiopia, Rwanda and Kenya as pilot studies. The “whole of society approach” endorsed by the CRRF, is “based on the Ugandan model” (Interview, INGO staff, Kampala, October 2020; see also ODI, 2019).

With frameworks and policies in abundance, a “bricolage of policy frameworks” (Hovil, 2018), the development-approach to refugee governance is instrumental to the Ugandan response. To date, the results of this approach are mixed: a recent report points out that 80% of refugees in Uganda live below the international poverty line (Hargrave et al., 2020). Whilst refugees are allowed to move around the country, they are only given assistance when they are in the settlements. Refugees who wish to leave have to request movement permits from administrators in the settlements and provide dates and reasons for travel. Some leave without permission. Refugees (not asylum seekers) are given an automatic work permit, namely a letter from the Office of the Prime Minister (OPM) in charge of refugee affairs, allowing them to work. Refugees are usually given a plot of land (though not the ownership), which they can farm themselves, with the idea that they will eventually become self-sufficient. Given the set-up, which prioritizes staying in the settlements, most refugees mainly work in the agricultural sector. In addition, a good number of refugees live outside the camps as self-settled in both rural and urban centres. For instance, while only about 5.6% of refugees and asylum seekers (80,506) are recognised as living in Kampala (UNHCR, 2020d), many more live as self-settled without assistance from UNHCR or OPM. Mainly employed in the urban informal sector, these urban refugees face their own challenges, having to provide for themselves (Bernstein & Okello, 2007). In sum, many refugees report frustrations and that their rights haven’t been implemented in practice (e.g. Ahimbisibwe, 2019).

In addition to these questions of livelihood and free movement, the Ugandan system is characterised by an overtly centralised system prone to local conflicts and over dependency on external donors. The latter links back to the question of how refugee protection is used as international leverage.

On the first point, the refugee system is deeply centralized to the OPM, who is in charge of most decisions, despite the fact that most refugees and all the settlements are in districts across the country. Moreover, certain parts of the country traditionally receive more development support related to the President’s personal connections (Hitchen, 2016; see also Hovil, 2018). A lack of decision-making and severe underfunding of districts and especially local municipalities makes refugee governance more complicated (see also Lozet & Easton-Calabria, 2020). Related are sporadic local conflicts between the local communities and refugee populations, especially in areas where access to resources and services such as healthcare and water are particularly scarce. Competition over natural resources including firewood, water and animal grazing rights as well as environmental degradation caused by the large number of refugees have become frequent (Coggio, 2018; Hargrave et al., 2020;ODI, 2019 ; Van Laer, 2019). Most recently, in September 2020, an estimated ten refugees were killed in clashes with the local population at a water point in Madi-Okollo, in Northern Uganda, and the Ugandan army was sent in to prevent further clashes (Okiror, 2020b). One problem is that the policy of land distribution uses community land for the refugees in Northern Uganda, whereas in South-Western Uganda it is government gazetted-land. As a result, the huge influx of refugees in recent years, particularly from Southern Sudan, as well as the increasingly protracted situation, has heightened tensions between refugees and hosts. Expectations of hosts benefiting from development funds for refugees have often stayed unfulfilled, which has led to increased frustrations (ODI, 2019; Van Laer, 2019).

On the second point: given that Uganda hosts one of the largest refugee populations in the world, it also depends on external donors. Despite this, a Solidarity Summit in 2017 raised only $ 350-million from the requested $2 billion (Ahimbisibwe, 2019). As of July 2020, Uganda had only 22% of the funding it needs to carry out its operation for the year (UNHCR, 2020b).^3^ Making the underfunding situation even more complicated, there is a fragile relationship to donors. In 2018, a corruption scandal was uncovered at the heart of UNHCR operations in conjunction with the OPM. This had two dimensions – firstly, an internal audit found that there was an overpayment in taxes ($10 million), contracts were awarded to ‘ghost’ contractors amongst other procurement irregularities as well as stockpiling of goods like sanitary pads and solar lamps to the tune of at least $10,000 (Parker, 2018). Some of the biggest donors threatened to withdraw their support, froze funding for some time, and the head of the UNHCR in Uganda was replaced (Coggio, 2018). Secondly, there was a problem in the counting of refugee numbers, leading to overblown figures. A biometric verification process showed that there was an excess of 300,000 refugees that did not actually exist (Schlindwein, 2018). One settlement even overestimated its number by 58% (Coggio, 2018).

The fallout from this experience still persists – though a number of officials from the OPM were fired as a result, no prosecutions or further investigation have followed, and some have long been reinstated. The situation highlights one persistent role for the Ugandan government – their (warranted) image as a refugee-friendly country of ‘open doors’ is also necessary in order to address their funding gap (see also Hargrave et al., 2020). Others have noted that their image also helps to turn away international attention from political oppression and persecution of minorities, such as from the LGBTQ+-community (Hitchen, 2016; Hovil, 2018) and perhaps more recently journalists and opposition leaders (United Nations, 2020).

Having discussed the characteristics of the refugee governance systems in South Africa and Uganda respectively, we now turn to their responses in light of the ongoing pandemic. In the following, we will discuss how Uganda and South Africa have responded to the pandemic, including with regards to new arrivals and borders, social protection and refugee-related rhetoric. We will then discuss the differences between the two countries and what this means for internal and external leverage in a final section.

### Pandemic responses in South Africa

The South African response in terms of refugees and asylum seekers has been very much a business-as-usual approach, with a securitized approach towards land borders, at least initially, limited access to social protection measures for asylum seekers and xenophobic messaging during the pandemic.

#### Borders and movement

South Africa was one of the first countries on the African continent to impose a hard lockdown in response to the COVID-19 pandemic, as noted previously. Amongst the measures implemented by the government were restrictions on local and interprovincial travel, with only essential service workers allowed to go to work outside their homes. Restrictions were also imposed on international travel, with only returning citizens and residents allowed entry into the country through monitored repatriation flights and under strict conditions which included a mandatory 14-day quarantine period on arrival. Those intending to return to their countries of origin were allowed to do so in coordination with their embassies in South Africa and through organised repatriation flights. As for asylum seekers, refugees and migrants, the mobility restrictions meant that no new asylum claims could be made since refugee reception offices closed immediately.^4^ Stopping regional migration was prioritised, despite no public health reasons to do so and deportations continued throughout the lockdown period. Finally, there was a lack of coordination on a regional level for example through the Southern African Development Community (SADC). These elements will be further detailed next.

First, the international travel restrictions and the closure of refugee reception offices also meant that there was no legal avenue for new asylum applicants on their way to South Africa and those who had already arrived but were still to lodge their applications to proceed. Asylum seekers from countries such as Ethiopia and Somalia travel long distances and sometimes take months to reach South Africa. Moreover, it is plausible that – also due to the porous borders – that new asylum seekers will have entered the country during the lockdown period. Because they need a transit visa to then get a section 22 permit and the RROs are closed anyway, asylum seekers who may have arrived during the lockdown and shortly before it commenced have been effectively rendered irregular and liable to arrest and deportation.

Second, the bureaucratized nature of the system, that doesn’t work with exceptions, and the undesirable nature of movement from the country’s neighbours, which has little to do with public health measures, is also shown through regional migrants who tried to return home. Some migrants, particularly from Zimbabwe took up the option to be repatriated back to their home country. However, there were newspaper reports that upon exiting the border, those that had overstayed their visas, not least due to the lockdown, were declared undesirable and banned from returning to South Africa for 5 years (Du Plessis, 2020). Even though the department of home affairs sought to rectify the situation and clarify that this was a system error (see Parliamentary Monitoring Group, 2020), it shows a priority in trying to undermine regional migration.

**Fig. 1:**
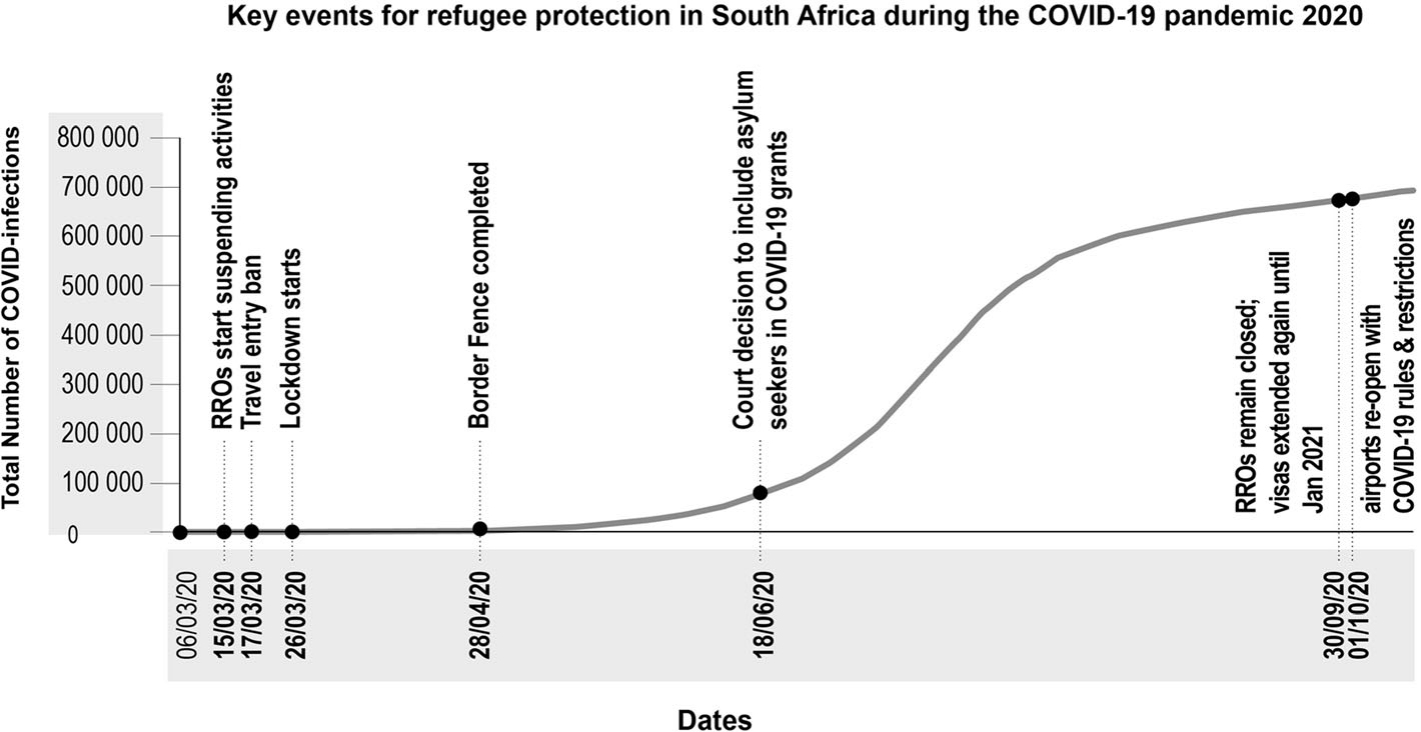
Key events for refugee protection in South Africa during the COVID-19 pandemic 2020. Source for COVID-19 infections: (Roser et al., 2020)

Stopping regional migration continued as an important trope during the pandemic. As part of the very first measures to deal with the pandemic, the government announced the building of a 40 km fence on the border between South Africa and Zimbabwe, with the minister of public works, Ms. Patricia de Lille justifying it as necessary to stop infected people from crossing the border, see also Fig. 1 below (Business Tech, 2020). The building of the fence is now the subject of a criminal investigation due to allegations of corruption in addition to allegations that the government paid almost double the price for a sub-standard product described as not fit for purpose (e.g. Felix, 2020). The minister of defence, Nosiviwe Mapisa-Nqakula has admitted that the fence is useless, highlighting that, “the whole thing of the fence has not worked and probably will not work. It doesn’t matter the quality of the fence we put up” (Ndenze, 2020). The securitised agenda is not lost however, as the minister told parliament that her department was considering deploying drones and other technologies to secure the border (Ibid.).

Similarly, whilst many countries across the world at the very least temporarily halted deportations (see Guadagno, 2020), South Africa continued to do so. The South African government deported a total of 1, 376 persons to neighbouring countries during the first lockdown period (Migration and Health Project Southern Africa, 2020). These included 705 Zimbabweans, 488 Mozambicans, 178 Basotho and 5 from Eswatini (see Home Affairs Portfolio Committee, 2020). Deportations during a pandemic may at the very least have exposed the deportees to COVID-19 infections during the process and also on arrival in their countries of origin.

Both these actions – deportations and fence-building - speak volumes to the externalisation approach of South Africa that contrasted public health reasons. They happened at a time when South Africa had more infections than any other country on the African continent (Zanker & Moyo, 2020). In a recent address, the minister of health acknowledged that South Africa accounted for 90% of the reported cases and 91% of deaths within the SADC region (Mkhize, 2020). The focus on the regional borders was at best misplaced, given the fact that at the time, the majority of infections had come through travellers from Europe rather than the neighbouring countries.

Finally, these actions also underlined the lack of coordination with neighbouring countries, where it would be expected that SADC countries would have a uniform strategy on the closing and reopening of borders, since borders are porous, and no amount of fence building can stop the movement of people to and from South Africa.

#### Social protection of refugees

The measures implemented by the government have had an adverse effect on the economy and the latest figures by the country’s statistical agency show that as much as 2.2 million jobs were lost due to the first lockdown (Statistics South Africa, 2020). Amongst the most stringent measures were the implementation of a curfew, the imposition of a number of bans - on the sale of alcohol and cigarettes or on large social gatherings such as sporting events and musical concerts - funeral gatherings were restricted to a maximum of 50 people and schools were temporarily closed, just as hotels and restaurants.

With many refugees and migrants working in the informal sector and the service industries, the lockdown had a huge impact on their ability to earn a living. In response to the widespread economic hardships caused by the lockdown, the South African government put in place social relief programmes such as the COVID-19 Social Relief of Distress Grant (COVID-19 SRD grant). Access continued the usual pattern of excluding migrants and asylum seekers from state benefits at a time when they were equally affected by the pandemic. Only refugees with section 24 permits and permanent residents were eligible to receive the COVID-19 SRD grant. It took a court decision on 18th June 2020 for the government to cater for asylum seekers with section 22 permits and migrants from Zimbabwe, Lesotho and Angola on Special permits.^5^ In the intervening period asylum seekers and migrants on special permits were excluded, and thus dependent on NGOs to provide basic necessities. There was no specialised aid for refugees in the initial pandemic period apart from UNHCR’s supported soap distribution to 16,190 people from the refugee and host communities in KwaZulu Natal Province (2020e).

Moreover, asylum seekers depend on their permits for everything, including banking, being able to attend school and working. They can only get their permits from the RROs. But some of these refugee reception offices already stopped providing their services even before the announcement of the lockdown, arguing that they needed to receive personal protective equipment (PPE) first (Bornman, 2020b). The department of home affairs later announced a blanket extension of the validity of asylum and refugee permits that expired during the period of the lockdown, which has been extended on several occasions, currently to the 30th March 2021. The extensions included permits and visas for migrants. However, the department has not clarified how it intends to deal with the backlog of extensions when it eventually opens its offices for applications, especially considering that it already has a historic backlog on its system. It also stands in contrast to other civic services which have resumed. Other civil services such as registrations of births and death were available from the end of March already.

#### Rhetorical response

There has also been a noticeable tendency of trying to use the pandemic as an opportunity to implement already-aspired for exclusionary policies that serve domestic interests.

While outlining her ministry’s response to the state of disaster at the beginning of the first wave, the Minister of Small Business Development, Khumbudzo Ntshavheni specified that, only South African owned and operated spaza shops were allowed to open during the lockdown. Spaza shops are small informal food stores found in township areas. Estimates count up to 100,000 across South Africa, with the majority run by migrants and refugees. As a result of this pronouncement, migrant-operated spaza shops were being forcibly shutdown by police on the first day of lockdown in some parts of the country (e.g. Zanker & Moyo, 2020).

These exclusionary regulations were rescinded after a few days, but the stance underlined the xenophobic nature of government decision-making in terms of separating between foreigners and citizens. The exclusion of the foreign-owned spaza shops from operating actually was prejudicial to South African citizens themselves who rely on these shops for their daily essentials and it meant that they had to travel longer distances to South African owned shops and therefore risking exposure to infection. It was thus a counterproductive strategy. More recently, the Finance Minister has highlighted that the post COVID-19 economy must prioritise South Africans over foreigners for jobs (Kwinana, 2020).

This rhetoric reveals the penchant of the South African government to fall back on a xenophobic agenda even during the pandemic.

### Pandemic responses in Uganda

Uganda is among the countries that instituted lockdown measures long before it registered her first case of COVID-19, as introduced above. Implemented gradually, lockdown measures were not only informed by information available from the WHO but also Uganda’s experience with earlier epidemics, most notably Ebola. Measures included but were not limited to restrictions on mobility both in and from outside the country and public gatherings. During the first 6 month of the pandemic, Kyangwali refugee settlement in the western district of Kikuube - home to 12,000 refugees - was under complete lockdown, after nearly 100 aid workers and refugees tested positive for COVID-19 (D’Orsi, 2020). Uganda’s response, though quick and decisive, by restricting movement and social interactions, has negatively impacted the social protection for refugees. Moreover, the opening of its borders during the pandemic highlighted the generosity of the Ugandan refugee system, though tied to grave funding gaps.

#### Borders and movement

Following what was happening elsewhere globally, attention first focused on limiting the mixing of people – those that had entered the country after the breakout of the pandemic and the rest of the population. A two-pronged approach was used to contain the spread, that is, restrictions on mobility and mixing of people. Travel restrictions were imposed on persons coming into the country such as mandatory quarantine in designated hotels and schools until proven to be free of the virus. These were later followed by a complete closure on national borders on Sunday 22nd March 2020, save for vehicles carrying cargo.

The greatest risk factor for COVID-19 remains the movement across borders into the country. Borders in Uganda have been a site of contention in relation to intra-regional mobility and trade. Porous borders across the region pose a risk factor for Uganda. According to the Assistant Chief Administrative Officer for Yumbe District, which shares a border with South Sudan, free movement of refugees across the borders poses a serious danger for COVID-19 transmission. “Refugees are free to move in and out of the country through the border points. It is impossible to police each crossing point. It is such a loophole that endangers us to COVID-19” (Interview, Yumbe, September 2020).

Whilst on March 25th 2020, the government officially closed the asylum space, Uganda made international headlines in July, by temporarily suspending the border closures to allow entry of refugees from DRC stranded close to the border. This opening in the middle of a pandemic was no doubt a difficult decision due to the public health ramifications it could hold. These fears were exacerbated by the fact that DRC and South Sudan, the major sources of refugees to Uganda, though hard hit by the pandemic did not have in place measures to contain the pandemic as compared to Uganda.

Nevertheless, on 1st July 2020, Uganda temporarily re-opened two border crossing points, in Gulajo and Mount Zeu in Zombo district, to asylum-seekers from the DRC. These were part of a larger group of over 45,000 people who had attempted to flee towards the Ugandan border with the DRC shortly after deadly militia attacks on civilians in Ituri province on 17th and 18th May 2020. According to the UNHCR, between 1 and 3 July, 3056 people entered Uganda, with children accounting for 65% percent of the group (UNHCR, 2020c). After a rigorous security and health screening at the border, the new arrivals were relocated to Zeu Farm Institute. The Ministry of Health and the district local government upgraded Zeu Farm Institute to serve as a quarantine and reception facility, installing 318 family tents, health screening areas, toilets, handwashing facilities and nine 10.000-l water tanks (UNHCR, 2020c). Later relocation of the refugees led to tension with local leaders who wanted the refugees to stay in the area and saw their move as a way to divide the community that lives both in the DRC and Uganda (Daily Monitor, 2020).

**Fig. 2:**
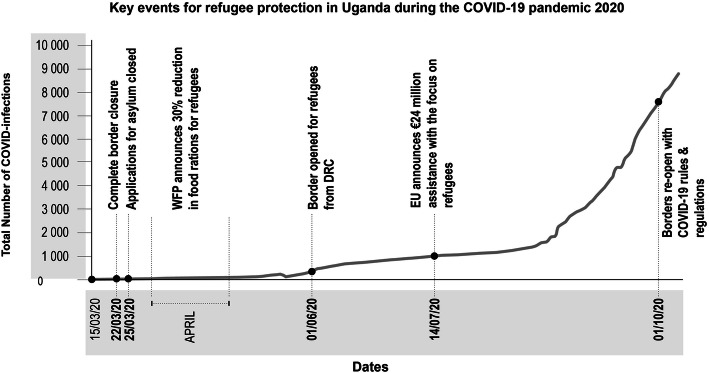
Key events for refugee protection in Uganda during the COVID-19 pandemic 2020. Source for COVID-19 infections: (Roser et al., 2020)

Though applauded for having a very favourable policy towards refugees and asylum seekers, with the pandemic wreaking havoc globally, the country found itself in an uncertain situation. At the very least, the move reiterated the positive image of the country, and therefore was a “diplomatic coup” (Interview, Civil Society Activist, digitally, October 2020) for the country, in dire need for new funds. Funding shortfalls and the disruption of global supply chains for relief food has negatively impacted refugee communities. As previously noted, as of July 2020, Uganda had only 22% of the funding it needs to carry out its operations for the year (UNHCR, 2020b). Due to insufficient funds, with a shortfall of $137million, the World Food Program also announced a 30% reduction in food relief in April (Okiror, 2020a). Funding shortfalls have inevitably forced reprioritization of activities including a reduction in food rations for the refugees. Refugees in Uganda had their food rations reduced from 12 Kgs per person in a household to 8Kgs per month. The result has been that some families have to go without food while others are forced into casual work to make ends meet. Humanitarian programs were scaled back as NGOs sought a no cost extension to activities initiated before the pandemic. Shortly after the border-opening, on the 14th July, the EU announced another €24 million in humanitarian assistance for the most vulnerable in Uganda with a special focus on refugees and their host communities (European Commission, 2020), see Fig. 2 below.

### Social protection of refugees

In addition to the direct cut in aid, the social economic impact of the pandemic takes a heavy toll on refugees. Particularly affected are the urban refugees, because the majority are employed within the informal sector with limited access to social protection, safety nets and are outside the scope of fiscal measures to stimulate the economy, which do not address the informal sector. In addition, Standard Operation Procedures to counter the pandemic have increased the cost of doing business because the onus is on business owners to put in place facilities for hand washing, temperature guns as well as ensure and enforce social distancing in their areas of operation. Not only are the handwashing facilities difficult to come by, but restrictions on mass gatherings are hindering their access to markets and informal sector work. Social distancing measures are cutting refugees off crowded spaces such as markets and streets forcing them to look for alternative spaces for doing business. Lockdown measures did not have a homogeneous impact on food access across the country. While in Kampala the food markets remained open and even had surplus food, in Arua there was an overall reduction in the food supply which resulted in the inflation of food market prices (Lozet & Easton-Calabria, 2020).

Therefore, measures to contain COVID-19 have marginalized refugees economically, also including those in settlements, by denying them access to alternative livelihood sources from agriculture. Businesses in the agricultural sector, where the majority of the refugees are employed, experienced the largest decline in business activity, with 76% of the firms reporting severe decline and 12% reporting moderate decline (Economic Policy Research Centre, 2020). Restrictions on freedom of movement make it particularly difficult for refugees to find work outside the settlements.

Whereas food markets nationally and within the settlements, remained open during the lockdown, this did not translate into access to food. With no money - since the population was not earning an income and had limited savings - not many were able to access food, including many refugees and asylum seekers. Reduction in business activity also resulted in a decline in access to work as employers either laid off staff or stopped recruiting. Restrictions on the labour market have affected refugees both directly and indirectly. Directly through not gaining employment. For example, graduates of vocational skills programs in Arua found it difficult to find new work assignments such as in the hotel and hospitality sector. Only graduates of tailoring were at an advantage since their skill was required in the making of face masks (Interview, Enabel programme officer, Arua, September 2020). Indirectly, refugees have also been affected as enterprises have laid off workers who have returned to their home regions and reclaimed their land from the refugees for own use.

Measures aimed at economic empowerment of refugees remain limited. While several economic stimulus measures have been proposed by development partners such as the European Union, the World Bank and the International Monetary Fund, these are targeting business owners and are not specific to refugees. The new EU fund, see above, was yet to materialize and start implementation at the time of writing.

Social protection, especially social assistance, remains uncertain for refugees in both settlements and urban areas. A reprioritisation of humanitarian assistance amidst funding short falls, has left refugees in a particularly precarious situation during the COVID-19 pandemic.

### Rhetoric around refugee protection in the COVID-19 response

COVID-19 in Uganda is shrouded in misbelief about its existence, effects and treatment. Until today, there is a section of the population that questions the existence of COVID-19 in the country, with support mainly observed on social media platforms (Amuge, 2020). The population’s complacency towards COVID-19 is partly blamed on the economic stress of the prolonged lockdown, the onset of political campaigns in the wake of elections in 2021, and some political leaders contravening the guidelines they have set in their interactions with their constituencies. The misbelief was also largely a consequence of how the government handled the pandemic. At the frontline of the fight were not health workers but security forces. These were used to enforce the presidential directives on COVID-19 resulting in the arrest, imprisonment and separation of families. In addition, several laws were hastily passed under the public health act to provide a legal basis for some of the draconian measures put in place, in part also as a precursor to the restrictive measures later applied during the elections.

Moreover, enhancing the mobility of District Security Officers instead of health teams and inaction on truck drivers carrying cargo into the country as the main importers of the virus into the country was further evidence of government misplaced priorities in the COVID-19 response. This was not helped by the large appetite for borrowable funds from international financial institutions. With a history of financial misappropriations, the population was not convinced of the government’s intentions (Cotterill et al., 2020).

The public perception of government misplaced priorities was extended to the decision to reopen borders to allow stranded refugees from the DRC to enter the country. This entry into Uganda was explained as a humanitarian act and was witnessed by two cabinet ministers, the Commissioner for Refugees and the UNHCR country representative. In spite of this political show, a section of Ugandans questioned the efficacy of opening up borders at a time when the country was at risk of importing the virus, accusing the government of putting ‘financial gains’ above the safety of Ugandans (Were, 2020). This was a small section of society however. On the whole, the country has been focused on the many dimensions of the COVID-19 economic fallout, with little public discourse on refugees in particular.

### Internal and external political gains from COVID-responses

The practice of refugee protection does not happen in a political vacuum, and the pandemic-response in the two countries highlights the political goals for certain refugee approaches, aimed at both domestic and international audiences. These will be discussed after looking at some of the commonalities and differences in Uganda and South Africa’s pandemic responses.

Both South Africa and Uganda have sizeable numbers of refugees and asylum seekers and laudable refugee policies. As destination countries, the economic pull in South Africa and the hospitality in Uganda cannot be ignored as key pull factors for refugees and migrants. Moreover, both countries surrounded by conflict-prone neighbours and thus serve as critical destinations for refugees. Having favourable policies towards refugees is vital as to not lock out those in need of help, nevertheless, practice is designed in such a way as not to encourage immigration.

Furthermore, both countries were quick to institute lockdown measures to contain the COVID-19 pandemic. Both countries reacted quickly, and during the first 6 months of the pandemic either managed to keep down the infections and COVID-19 deaths (Uganda) or to flatten the curve after the first wave of infections (South Africa). Lockdown measures by and large aimed at protecting the population especially the most vulnerable ones from the pandemic. However, lockdown measures were also sometimes used to as an opportunity to pass restrictive policies on people’s rights and freedoms. No wonder therefore that some of the population in both countries remained ambivalent of the measures put in place to control COVID-19.

There are also many differences between the countries. The different economic strengths of the two countries for example also shows in its pandemic response. Direct relief packages were available in South Africa – albeit belatedly for asylum seekers. This was not an option in Uganda, for anyone.

Uganda was quick to shut off all international travel – before officially recording their first case. It is of course much easier to do so, considering that the country has only ten entry points – including only one international airport – whereas South Africa has 72, including ten airports that serve as points of entry to the country. In South Africa, the focus seemed to be on stopping regional migration, though this was not supported by public health arguments, with the country having far more COVID-19 cases than its neighbouring countries. In Uganda, this was less of an issue – borders were even formally opened as discussed - though there were also concerns with truck drivers as potential vectors of the virus. The population remained suspicious of the government intentions- putting the economic goals over population’s health.

Moreover, both countries have their own specific characteristics of refugee protection, which are mirrored in their pandemic responses. In South Africa, there is a state-led bureaucratized merging of refugees and migrants, in the shadow of institutionalized xenophobia leading to increasing securitisation. During the pandemic, the response has been a securitized approach towards regional land borders, limited access to social protection measures for asylum seekers and several cases of xenophobic political rhetoric. The Ugandan system, though unique in size and approach, is characterised by an overtly centralised system prone to local conflicts and with a dependency on external donors. Uganda’s response, though quick and decisive, by restricting movement and social interactions, has negatively impacted the social protection for refugees. Moreover, the opening of its borders during the pandemic highlighted the generosity of their approach whilst reiterating their problematic funding gaps.

Broadly speaking, the pandemic responses towards asylum seekers and refugees align with wider goals of the two countries – gaining legitimacy from a frustrated public (South Africa) versus cementing a positive image for the international community in the wake of the 2021 elections (Uganda). In South Africa, the ruling party has received much criticism for the COVID-19 response, which has thrown millions of South Africans to the economic brink, in what was already a fragile situation. Corruption, poverty and inequality have long led to scapegoating of migrant communities in order to gain political leverage domestically (Moyo & Zanker, 2020). Therefore, South African politicians sought to continue to politically capitalize internally from reiterating a division between migrant and refugee communities as a threat to poor and disenfranchised South Africans. Whilst distress grants were retroactively granted for asylum seekers as well, the inability to renew permits shows the lack of priority in this area, jeopardising access to many additional services for asylum seekers.

Uganda on the other hand has much to gain from the positive image of refugee protection, in what has become a not always easy relationship to their humanitarian and development partners (see also Hovil, 2018). At the end of August 2020, the OPM in charge of refugee affairs, failed to renew permits for more than 200 refugee aid agencies. Though this was likely to only be temporary – many aid agencies were unable to renew their permits in Kampala due to restrictions on movement within the country – it also sends a clear signal to the international community, “that the country is struggling with its refugee burden” (D’Orsi, 2020). This “burden” cannot be taken on alone, and nothing was more effective at showing their pro-refugees stance than opening their borders to let in refugees despite the ongoing pandemic. The opening also distracted from other issues - such as the draconian measures which led to the deaths of at least 12 persons due to overzealous police officers enforcing lockdown measures (Atuhaire, 2020). Another key development was the run-up to the 2021 elections, which led to repressive crackdowns on opposition leaders and journalists (Human Rights Watch, 2020).

In sum, the pandemic responses speak to internal – South Africa – and external – Uganda- political gains. Of course, the South African government is also concerned with their external image, just as much as the Ugandan government is with their own voting populace. South Africa has received condemnation for not better controlling the outbreaks of xenophobia, including the recalling of the Nigerian ambassador in 2019 (Chigumadzi, 2019). In Uganda, the border opening was used in a tweet in 2021 to show loyalty to the disputed winner of the Presidential elections, Museveni, colloquially referred to as M7, with the hashtag #WhyUGDecidedM7 (Nazziwa Racheal, 2021). Nonetheless, in terms of refugee protection, the respective actions have distinctive overarching audiences. Namely, it comes down to who is watching: in Uganda – the international community – who would hopefully be shamed into digging deeper into their pockets. In South Africa, the impoverished communities who would be willing to buy into the argument of security threats and scapegoating efforts translating into votes at upcoming elections.

## Data Availability

Data sharing is not applicable to this article as no datasets were generated or analysed during the current study.

## Footnotes

1 Though this paper focuses on refugees, we acknowledge that a clear differentiation between refugees and migrants is at best analytically blurry and in most cases a political decision (e.g. Mourad & Norman, 2020). This is why we also refer to migrants and migration governance when necessary.

2 The project “Forced Displacement in Africa” is based at the Arnold Bergstraesser Institute at the University of Freiburg. For more on funding see Declarations.

3 The operational fund for UNHCR Southern Africa (which includes South Africa and neighbouring countries) also had a 97% funding gap by the end of 2020 (UNHCR, 2020f). Due to the different approach to hosting refugees in the communities themselves, there is less of a formal model of funding the livelihoods of refugee populations, who – at least on paper and excluding the most vulnerable populations – have to largely fend for themselves.

4 This stance has persisted for a year now.

5 The case was brought to court by the Scalabrini Centre of Capetown. The special permits for Zimbabweans and Lesotho nationals were introduced in 2010 and 2015 respectively in order to regularise undocumented migrants from the two countries and also relieve pressure on the asylum system in the case of Zimbabweans. Angolans were given special permits after the cessation of their refugee protection in 2013 (Carciotto & Mavura, 2016).
